# Structural classification of proteins and structural genomics: new insights into protein folding and evolution

**DOI:** 10.1107/S1744309110007177

**Published:** 2010-07-06

**Authors:** Antonina Andreeva, Alexey G. Murzin

**Affiliations:** a MRC Laboratory of Molecular Biology, Hills Road, Cambridge CB2 0QH, England

**Keywords:** structural genomics, protein folding, structural classification

## Abstract

During the past decade, the Protein Structure Initiative (PSI) centres have become major contributors of new families, superfamilies and folds to the Structural Classification of Proteins (SCOP) database. The PSI results have increased the diversity of protein structural space and accelerated our understanding of it. This review article surveys a selection of protein structures determined by the Joint Center for Structural Genomics (JCSG). It presents previously undescribed β-sheet architectures such as the double barrel and spiral β-roll and discusses new examples of unusual topologies and peculiar structural features observed in proteins characterized by the JCSG and other Structural Genomics centres.

## Abstract 2

This review article surveys the protein structures determined by Joint Center for Structural Genomics and published in this special issue of *Acta Crystallographica Section F.*

## 1. Introduction

The notion of protein structure classification emerged from several studies conducted during the late 1970s and early 1980s that aimed to elucidate the basic principles of protein folding and protein structure evolution. The early work of Chothia and coworkers pioneered the division of protein structures into four major classes based on their secondary-structure composition and demonstrated that simple geometrical features of secondary-structural elements govern their mutual arrangement in distinct architectures (Chothia, 1984 ▶; Chothia *et al.*, 1977 ▶; Levitt & Chothia, 1976 ▶). Later, Jane Richardson provided a more detailed classification deduced from the topological details of less than 200 structures (Richardson, 1977 ▶, 1981 ▶). The progress made in the field in the 1980s was reviewed by Chothia & Finkelstein (1990 ▶). By that time, the term ‘fold’ was already established and it was intended to outline three major aspects of protein three-dimensional structure: the secondary structures of which the protein is composed, their relative arrangement and the path taken through the structure by the polypeptide chain. Thus, the fold of a protein was defined through its composition, architecture and topology.

It also became apparent that homologous proteins of similar sequence adopt the same fold. It was also noted that some folds are populated by proteins with dissimilar sequences. These observations raised the question of whether the structural similarities between different proteins indicate distant homology or simply result from the basic principles of physics and chemistry. The Structural Classification of Proteins (SCOP) database, established in 1994, circumvented this argument by introducing a new category: the superfamily. This level of classification aimed to group together proteins that have probably descended from a common ancestor but whose sequences have diverged beyond detectable similarity (Murzin *et al.*, 1995 ▶). The notion of probable distant homology ‘relaxed’ the definition of protein fold by transforming it into a ‘consensus’ fold common to a set of evolutionarily related proteins. Dissecting the protein structure into an evolutionarily conserved core and a variable periphery kept the discrete classification, thus avoiding the continuous folding space and Russian-doll problems that arise from partial structural similarities.

It was thought at that time that the number of architectural types was limited. Moreover, although some structural variations were observed amongst evolutionarily related proteins, none of these affected the common structural core. Therefore, it was assumed that the protein fold is evolutionarily stable in that it retains its characteristic features, although some structural changes could be anticipated. Similarly, it was thought that every protein folds into a single three-dimensional structure and that its structural core is insensitive to large conformational changes related to function or formation of quaternary structure.

At the advent of structural genomics (SG) and the Protein Structure Initiative (PSI; http://www.nigms.nih.gov/Initiatives/PSI/), analysis of the trends of newly discovered folds seemed to indicate that most of protein fold space had been explored. The growth in the number of new folds in SCOP had almost stalled and the rate of discovery of new superfamilies and families obviously slowed down. After the PSI launch the number of new folds, superfamilies and families rose again, mainly because the PSI SG centres targeted proteins with no significant sequence similarity to known structures. Currently, owing to the joint efforts of SG centres and independent structural biology groups there is more than one structural representative for most of the characterized families (Andreeva *et al.*, 2008 ▶). Recent analysis of the distribution of protein families characterized by structural genomics has confirmed the dominant role of the largest known superfamilies, which have grown further in their number of constituent families (Andreeva *et al.*, 2008 ▶). In addition, other superfamilies have grown large rather unexpectedly. The evolutionary success of these ‘new rich’ superfamilies is probably a consequence of the presence of unusual conserved and presumably functionally important features in their folds. One of these ‘new rich’ superfamilies, for example, is the dimeric α+β barrel superfamily in SCOP, several new members of which have come from the first structures of metagenomic sequences (Yooseph *et al.*, 2007 ▶).

Initially, it was anticipated that a large number of new folds would be discovered owing to the breath of coverage of fold space targeted by the PSI. Interestingly, this has not turned out to be the case as a substantial portion of the structures coming from SG revealed significant structural similarities to already known folds and in fact represent variations of existing protein architectures and topologies. However, there were several unexpected findings of previously unseen topologies and architectures. For instance, a number of SG structures revealed superfamily-specific folds in which the core β-­sheet structures are tailored into unique shapes. PSI also greatly increased the number of protein topologies with high contact order, which is known to limit the success of current *ab initio* structure-prediction methods (Bonneau *et al.*, 2002 ▶), thus providing invaluable high-resolution templates for modelling. Without previous pre­conceptions, comparisons of some SG structures revealed dramatic structural variations in related proteins that go beyond the expectations based on their sequence similarity. These provide convincing examples of how protein folds can evolve without compromising the integrity of the structure of the functional site.

The plethora of structural data delivered over the past decade by SG and independent groups revealed numerous examples of atypical structural features and structural variations that have challenged many longstanding tenets in protein science (Andreeva *et al.*, 2007 ▶; Andreeva & Murzin, 2006 ▶). Amongst these, for instance, is the discovery of the deep trefoil knot (Nureki *et al.*, 2002 ▶). SG has determined the structures of several knotted proteins, which in turn helped to dispel one of the oldest dogmas in molecular biology prohibiting knots in protein chains. In addition, the old folding paradigm ‘one sequence – one structure’ is increasingly being challenged as more and more structural variations are observed in protein families and their individual members. It has become evident that the protein fold is neither physically nor evolutionarily invariant (Andreeva & Murzin, 2006 ▶).

These structural variations found amongst homologous as well as individual proteins create nontrivial structural relationships at any ‘evolutionary’ level of SCOP and increase the structural diversity within families and superfamilies. In essence, the classification of a new protein in SCOP depends on its relationship to protein(s) of known structure. If there is strong evidence that a protein is homologous to other protein(s) in SCOP then it is classified into an existing superfamily. The fold of these evolutionary-related proteins is an attribute that describes the given evolutionary lineage. If a protein is not homologous to any protein in SCOP and has a fold that differs from any known fold in composition, architecture or topology, then it is classified as a novel fold. Paradoxically, a protein with a novel fold may well be structurally similar to (but still distinct from) previously classified proteins, whereas a protein classified as a new member of an existing superfamily may display novel features in architecture or topology.

**Figure 1:**
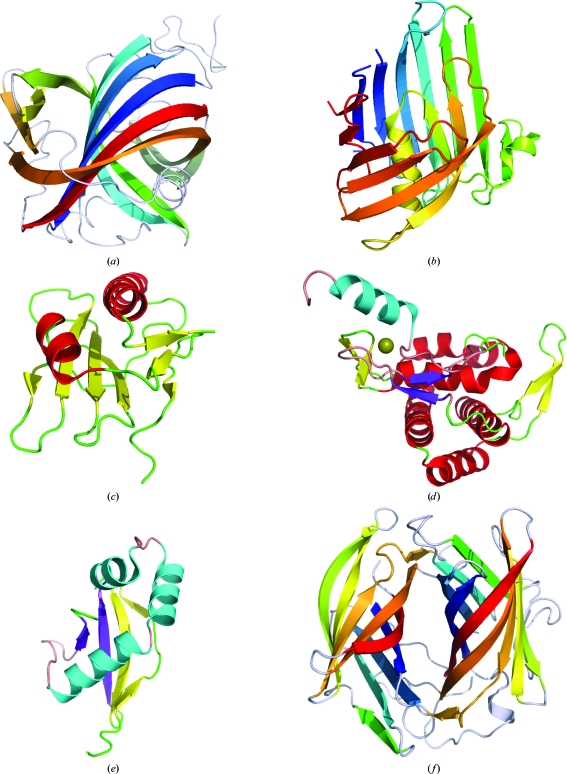
Gallery of selected protein structures determined by the JCSG (see also Figs. 5 ▶, 6 ▶ and 7 ▶). (*a*) Acetoacetate decarboxylase (ADC) subunit (PBD entry 3c8w). β-Strands in the double-barrel β-sheet are shown as coloured arrows; other secondary-structure elements and loops are shown as silver coils. (*b*) DUF1089 protein PA1994 (PDB entry 2h1t) coloured by rainbow. An ‘unswapped’ monomer is shown, a large β-sheet of which is folded into a spiral roll. (*c*) DUF1831 protein lp2179 (PDB entry 2iay) coloured by secondary structure: red, α-helix; yellow, β-strand; green, loop. (*d*) DUF1470 protein Jann2411 (PDB entry 3h0n) coloured by secondary structure, with the N-terminal subdomain coloured as in (*c*) and the C-terminal subdomain coloured using an alternative palette: cyan, α-helix; purple, β-strand; pink, loop. The sphere represents the zinc ion. (*e*) DUF1488 protein Shew3726 (PDB entry 2gpi) coloured by secondary structure, with the rare left-handed β-X-β unit coloured using an alternative palette. (*f*) DUF2006 protein NE1406 (PDB entry 2ich) viewed along the pseudo-twofold axis that relates its similar barrel domains. The topologically equivalent β-strands in both domains and in ADC (*a*) are shown in the same colour.

In this article, we survey protein structures resulting from various SG efforts, in particular proteins structurally characterized by the Joint Center for Structural Genomics (JCSG) and published in this special issue of *Acta Crystallographica Section F* (Fig. 1 ▶ and other images below). Here, we focus on some interesting examples of novel protein architectures and unusual topologies that have given new insights into protein folds and evolution.

## 2. Novel architectures

The main building blocks of protein folds are α-helices and β-sheets, which pack together enclosing clusters of nonpolar residues or hydrophobic cores. In theory, the number of different possible globular arrangements of these blocks around a single hydrophobic core is limited. All of the simple ‘two-layer’ architectures have already been observed (Chothia *et al.*, 1997 ▶). Nevertheless, new architectural types are still being uncovered. Typically, these new architectures are multilayer and/or made of customized building blocks such as nonpolar helices that can be buried in the protein interior, combinations of long and short helices, β-sheets that are tailored into particular shapes *etc*. Such customized secondary structures are usually coded by particular or atypical sequence patterns, which tend to be superfamily-specific.

**Figure 2:**
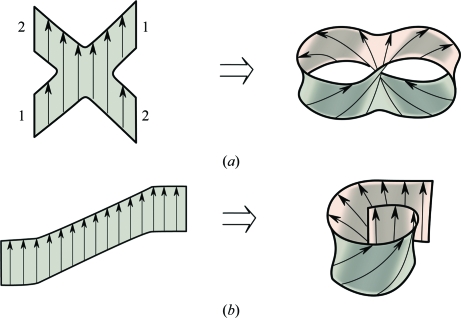
New β-sheet architectures. (*a*) A bifurcated X-shaped β-sheet can fold upon itself on both sides, forming a double barrel. (*b*) A very large β-sheet can be folded into a β-spiral roll with overlapping edges. This architecture combines features of both β-­barrel and β-sandwich. In both parts, for simplicity, the arrows denote β-strands but do not define the strand directionality. The actual β-sheets of these architectures may comprise parallel and antiparallel strands.

The β-sheet is a very versatile building block and comes in many different shapes and sizes. In addition to the two major architectural types of β-sheet proteins, β-sandwich and β-barrel, a number of minor types have been discovered, including β-helices, β-propellers, β-prisms, β-clips, barrel–sandwich hybrids *etc.* (Chothia & Murzin, 1993 ▶). Below, we describe two new β-sheet architectures found in structures determined by the JCSG (Fig. 2 ▶).

### 2.1. ‘Double-barrel’ fold: a new multi-barrel architecture of acetoacetate decarboxylase

The β-sheet barrel is a major architectural type (Murzin *et al.*, 1994 ▶). It is formed by a staggered β-sheet that adopts a hyperboloid-like shape with saddle-like sides. Owing to the stagger of its strands, there are free strand edges at the barrel ends to which additional β-­strands can attach. Theoretically, by addition of a few extra strands to both ends on the same side of the barrel a second conjoined barrel can be formed. The resulting double barrel will have a bifurcated β-­sheet with an X-like shape, the opposite edges of which are joined together at the either side of the β-sheet, as shown schematically in Fig. 2 ▶(*a*). Multibarrel β-sheets have previously only been observed in some oligomeric structures, but recently this architecture has been discovered in the subunit fold of acetoacetate decarboxylase.

The structures of three members of the acetoacetate decarboxylase family have been determined: two of them by the JCSG [PDB entries 3c8w (JCSG, unpublished work; Fig. 1 ▶ *a*) and 3cmb (JCSG, unpublished work)] and one by another group (PDB entry 3bh2; Ho *et al.*, 2009 ▶). They form different oligomers, dodecameric (3c8w and 3bh2) and tetrameric (3cmb), the constituent dimers of which are very similar. The subunit fold of these oligomers comprises two conjoined barrels capped by helices. One of the barrels has a round shape and consists of seven strands, whereas the other is flattened and contains nine strands. The flattened barrel is structurally similar to the barrel repeats of the AttH-like fold of DUF2006 (PDB entry 2ich; see below). The interior of the rounded barrel contains the active-site channel. This channel could have evolved from an open binding site located on the surface of a single barrel with a simple meander topology. This binding site could have been enclosed into the second barrel by the addition of a few extra β-strands in the connecting loops between the strands of the first barrel.

### 2.2. Spiral β-roll: a new architecture that might have evolved from known folds

A novel type of β-sheet architecture, the so-called spiral β-roll, has been found in the structure of the first representative of DUF1089 (PDB entry 2h1t; Fig. 1 ▶ *b*; Bakolitsa, Kumar, McMullan *et al.*, 2010 ▶). The 2h1t structure is a strand-swapped dimer. Here, we consider the fold of the ‘unswapped’ monomer that includes the N-terminal strand of the adjacent subunit instead of the equivalent strand in its own chain. This fold has a large predominantly antiparallel β-sheet of 15 strands. The central part of the β-sheet curves in a similar way as a wide β-barrel, whereas its edges overlap in a sandwich-like fashion, as shown schematically in Fig. 2 ▶(*b*). This overlap contains three strands at one edge, five strands at the other and a single α-helix trapped inside. The helix and the innermost strand correspond to the most conserved region in the sequence alignment of the DUF1089 family.

Several proteins in SCOP have somewhat smaller β-sheets (11–13 strands) but a similar topology to the DUF1089 sheet (Hirano *et al.*, 2008 ▶). Despite the fact that the shapes and curvatures of these sheets vary greatly from an open sheet to a nearly complete barrel, there is growing evidence that many, if not all, could be distantly related. Further analysis of this structural class may eventually result in the unification of these families and DUF1089 into a novel superfamily in SCOP. In this case, the unusual spiral β-roll fold would become an attribute of this particular family.

### 2.3. Distinct architecture with similarity to known folds

The representative structure of DUF1831 (PDB entry 2iay; Fig. 1 ▶ *c*) has extensive similarity to many members of the TATA-binding protein-like (TBP-like) fold in SCOP (Bakolitsa, Kumar, Carlton *et al.*, 2010 ▶). The presence of two β-sheets in its fold makes it distinct from the TBP-like fold, which is based on a single β-sheet. The additional β-­sheet comprises the first two N-terminal strands and the C-terminal strand. The two sheets come together as two walls at the corner and accommodate two helices in between. The sequences coding for the additional β-sheet are present and fairly conserved in all family members. In addition, DUF1831 appears to be unrelated to any known member of the TBP-like fold. There is no detectable sequence similarity between DUF1831 and any of the superfamilies of this fold. Neither of these superfamilies possesses a functional site in an equivalent topological location to the cluster of conserved surface residues (on the helical side) of the DUF1831 fold. Taking all this into account, the 2iay structure defines a new fold in SCOP.

### 2.4. A new architecture with a family-specific fold

The representative structure of DUF1470 (PDB entry 3h0n; Fig. 1 ▶ *d*) can be divided into two subdomains (Bakolitsa, Bateman *et al.*, 2010 ▶). The structure of the larger N-terminal subdomain shows no overall similarity to any known structure. It is mostly α-helical with two β-hairpins that stick out and do not contribute to the protein core. Three of its core helices form an up-and-down bundle, against one side of which the remaining helices and the C-terminal sub­domain are packed. The structure of the C-terminal subdomain resembles the treble-clef fold (Grishin, 2001*b* ▶) of the glucocorticoid receptor-like superfamily of zinc fingers (Murzin *et al.*, 1995 ▶). It does bind metal (zinc) ion, but it has a distinct metal-binding motif consisting of four invariant cysteine residues.

The residues at the interface between the two subdomains of DUF1470 are fairly well conserved within the family, as are the surface residues of both subdomains near the interface. The tight association of the two subdomains may be essential for function and would therefore be conserved. Thus, the representative structure of DUF1470 defines a novel family-specific fold in SCOP.

## 3. Unusual topologies

Several empirical rules regarding protein topology have been established by previous analyses of protein structures. One of them postulates that secondary-structure elements that are adjacent in sequence are adjacent in the structure. That is, protein structures generally have a low contact order (Bonneau *et al.*, 2002 ▶). Other rules describe topological preferences, such as the absence of knots, the right-handedness of connections between two parallel β-strands *etc*. (Chothia & Finkelstein, 1990 ▶; Sternberg & Thornton, 1976 ▶, 1977 ▶). These rules have been the subject of many studies looking for their physical and/or biological bases. The statistical stringency of these rules is now in need of revision owing to the recent influx of new protein structures. Moreover, many more known exceptions to these rules are found in recent protein structures with unusual topologies. Some of these are discussed in this section.

### 3.1. A new fold with a rare topological feature

The classification of the first representative of DUF1488 (PDB code 2gpi; Fig. 1 ▶ *e*) into a novel fold in SCOP was straightforward owing to the presence of a rare topological feature. The 2gpi structure has a simple α+β fold with a mixed four-stranded β-sheet (Han, Krishna *et al.*, 2010 ▶). A peculiar feature of this fold is the left-handed crossover connection between the last two strands that are parallel to each other.

Since the C-terminal β-strand sequence is highly conserved in the family alignment, the left-handed crossover is characteristic of the entire family. There is no structure in the database that displays partial similarity and contains this rare feature and therefore the fold of DUF1488 is considered to be distinct.

### 3.2. Knotted protein structures

**Figure 3:**
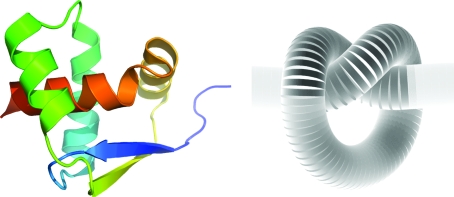
A trefoil knot in the structure of the uncharacterized protein MJ0366 (a duplicated RHH motif).

Until recently, deep knots had not been observed in protein chains and therefore their formation was considered to be impossible. In recent years, several structural genomics centres have determined structures of various knotted proteins, in particular proteins from a fast-growing superfamily of putative methyltransferases. All the members of this superfamily contain an obligatory deep trefoil knot that forms the binding site for an *S*-adenosylmethionine cofactor (Lim *et al.*, 2003 ▶). A different trefoil knot, the smallest of its kind, has been discovered in the structure of the uncharacterized protein MJ0366 (PDB entry 2efv; T. S. Kumarevel, P. Karthe, S. Kuramitsu & S. Yokoyama, unpublished work; Fig. 3 ▶). This protein is classified into the ribbon–helix–helix (RHH) superfamily of DNA-binding proteins in SCOP and it is very likely that it may exhibit a similar DNA-binding function. This knotted fold, comprising two RHH motifs arranged as in the typical RHH dimeric fold and connected by a linker, probably resulted from a gene-duplication/fusion event. Interestingly, a fully functional protein from the two linked RHH subunits of the Arc repressor was artificially created long before the discovery of the first deep knot (Robinson & Sauer, 1996 ▶), but it was not recognized at the time that the single-chain protein could potentially form a knotted fold.

### 3.3. Globular oligomers of noncompact subunits and high-contact-order monomeric structures

**Figure 4:**
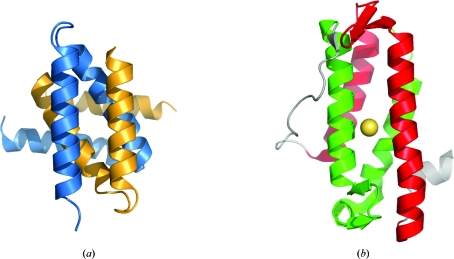
(*a*) The structure of UPF0352 protein CPS2611 (PDB entry 2ota; S. M. Vorobiev, W. Zhou, M. Su, J. Seetharaman, H. Wang, H. Janjua, K. Cunningham, L.-C. Ma, C. Liu, T. B. Acton, R. Xiao, G. T. Montelione, L. Tong & J. F. Hunt, unpublished work) is an example of an obligatory oligomer. It is composed of two interlocking noncompact subunits, which are coloured orange and blue. (*b*) A representative structure of the DinB-like family member (PDB entry 2f22; JCSG, unpublished work). It contains two interlocking structural repeats, which are shown in green and red.

It was assumed that in the beginning all proteins were monomeric and some then evolved into oligomeric structures. A very small number of known globular oligomers composed of interlocking noncompact subunits were considered to be an exception confirming this rule. In recent years, the PSI has contributed to the discovery of many new families, the individual members of which form this type of oligomer (Fig. 4 ▶ *a*). These include the following SCOP families: YejL-like (Pfam 07208; DUF1414), HP0242-like (Pfam 09442; DUF2018), YopT-like (Pfam 09467), AF2212-like (Pfam 01954; DUF104), YonK-­like (Pfam 09642), AF2331-like, SMc04008-like (Pfam 06844; DUF1244) and CsrA-like (Pfam 02599) (Murzin *et al.*, 1995 ▶). Interlaced obligatory oligomers have also been discovered in some new members of the previously defined superfamilies, for example in the structure of a putative all α-helical NTP pyrophosphohydrolase (PDB entry 2rfp) determined by the JCSG (Han, Elsliger *et al.*, 2010 ▶).

These new structural insights change our understanding of the formation and evolution of oligomeric structures. An evolutionary scenario in which monomers could evolve from oligomeric structures with a single hydrophobic core seems to be equally plausible. Indeed, many monomeric globular folds composed of structural repeats may have evolved from globular assemblies of single-repeat subunits by gene-duplication/fusion mechanisms. Ancestral non­globular interlocking subunits could result in protein structures of higher contact order, such as the representatives of the DinB-like family, as first characterized by the JCSG (Fig. 4 ▶ *b*).

## 4. Fold evolution

Nowadays, it is widely accepted that the protein fold can change and that these changes may affect not only the peripheral elements of the structure but also the core elements (Andreeva & Murzin, 2006 ▶; Grishin, 2001*a* ▶; Murzin, 1998 ▶).

**Figure 5:**
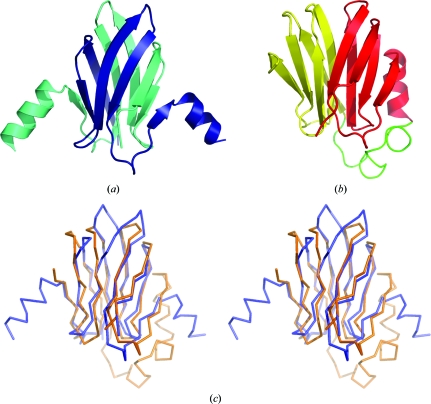
Structures of the CsrA dimer (*a*) and the C-terminal domain of YqeH (*b*). The individual subunits of CsrA are coloured cyan and blue, whereas the YqeH structural repeats are shown in yellow and red. (*c*) Stereoview of the superimposition of the CsrA dimer (blue) and the C-terminal domain of YqeH (orange).

An example is the recently discovered nontrivial structural relationship between the global regulatory protein CsrA (PDB entry 1vpz; Rife *et al.*, 2005 ▶) and the C-terminal domain of YqeH GTPase (PDB entry 3ec1; Sudhamsu *et al.*, 2008 ▶; Fig. 5 ▶). The CsrA structure, which was first determined by the JCSG, revealed a dimer of interlocked subunits forming a single domain with an atypical β-sandwich architecture. The YqeH-domain structure (3ec1) consists of two compact structural repeats organized into a β-sandwich fold of very similar architecture. The topologies of these two sandwiches are related by segment swapping. Interestingly, CsrA has recently been shown to bind RNA and it seems plausible that YqeH could also have this function. A hypothetical evolutionary link between the two domains would be a CsrA-like dimeric protein with compact subunits packed side-by-side like the YqeH repeats.

### 4.1. Two-domain fold with internal duplication

The fold of DUF2006 (PDB entry 2ich; Fig. 1 ▶ *f*) comprises two similar structural domains related by a pseudo-twofold axis (Chiu *et al.*, 2010 ▶). The presence of two structurally similar domains suggests the likelihood that a gene-duplication/fusion event has occurred during the evolution of this protein family. Each domain consists of a flattened open barrel. The N-terminal domain has an insertion of two additional β-strands. There is no significant sequence similarity between the constituent domains of 2ich. The most conserved residues are located at the domain interface and form a pocket. This strongly suggests that the DUF2006 two-domain fold is a single functional unit. Moreover, the structures of the constituent domains display no similarity to any other known barrel-like fold and therefore it was classified in SCOP as a single unit: the AttH-like fold.

### 4.2. Fold changes following a duplication/fusion event

**Figure 6:**
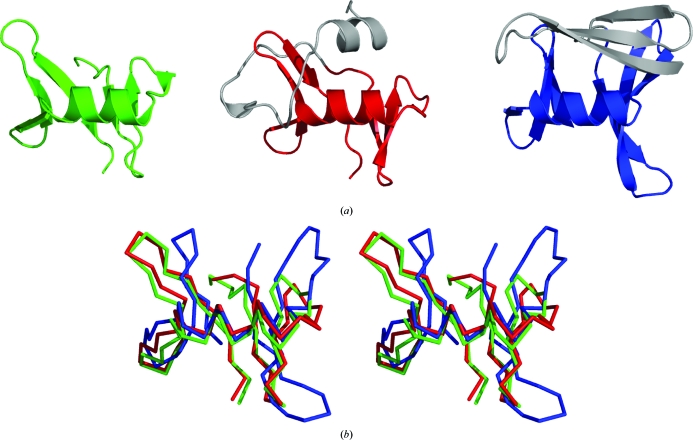
A consensus fold of the N- and C-terminal domains of the DUF1285 family. (*a*) Side-by-side comparison of the N-terminal domains of 2ra9 (green) and 2re3 (red) and the C-­terminal domain of 2re3 (blue). Nonconserved additional regions are shown in grey. (*b*) Stereoview of the superimposition of the common parts of the three domains.

The JCSG has determined the structures of two different members of the DUF1285 family (PDB entries 2re3 and 2ra9) with divergent sequences (Han, Bakolitsa *et al.*, 2010 ▶). Their comparison allows the identification of structurally conserved and variable regions and improves the initial classification. The 2re3/2ra9 fold can be divided into two structurally similar domains, the mutual arrangement of which is conserved owing to a tight association. The N-terminal domain is more conserved than the C-­terminal domain in both sequence and structure. The C-terminal domain of 2re3 contains a barrel-like β-sheet, resulting in some structural similarities to the PH-­like fold. The N-terminal domains of both 2re3 and 2ra9 show no global similarities to known protein structures, except for the structure of the C-terminal domain of 2re3. The consensus fold of these domains comprises an α-helix flanked at each end by a three-stranded meander β-sheet (Fig. 6 ▶). The presence of two copies of this unique fold in the same protein structure (2re3) suggests a gene-duplication/fusion event, analogous to 2ich and many other examples in SCOP. The ancestral structure probably contained a few additional structural elements at the N-terminus. These elements could have evolved into the N-terminal tail of the N-terminal domain or the barrel-like extension to the N-terminal β-sheet of the C-terminal domain, as observed in the 2re3 structure. During evolution, the C-­terminal β-­meander of the consensus fold, which is absent in the C-­terminal domain of 2ra9, was probably lost in this and some other structures. Recent analysis has suggested that DUF1285 belongs to a new superfamily that also includes the UPF0598 and PfamB PB002487 families (L. Aravind, personal communication; N. V. Grishin, personal communication; http://iole.swmed.edu/~grishin/2re3/2re3.htm). All three families are clearly taxa-specific: DUF1285 is found in α-, γ- and δ-­proteobacteria, PB002487 in β-proteobacteria and UPF0598 in Metazoa. The distinct phylogenetic distributions suggest that these families may have evolved different functional and structural features. Interestingly, a member of PB002487 has already been targeted for structure determination by the JCSG (Target ID 392148, gi:91785099).

## 5. Metamorphic proteins

A small but growing number of ‘metamorphic’ proteins adopt different folded conformations for the same amino-acid sequence under native conditions (Murzin, 2008 ▶). Unlike prions, they undergo reversible conformational changes. The recent discoveries of metamorphic proteins that are capable of independent interconversion and of an abrupt fold change in a protein lineage suggest a general nature for this phenomenon.

**Figure 7:**
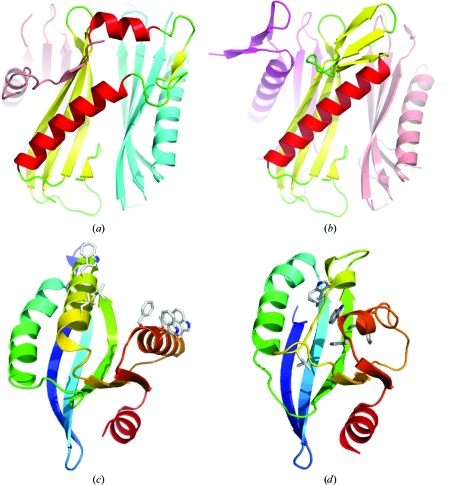
Metamorphic proteins.  (*a*, *b*)Side-by-side comparison of alternatively folded subunits of the DUF74 pentamer (PDB entry 1vr4). Chain *A* and chain *D* (*b*) are coloured according to their secondary structure. The adjacent subunits are coloured as follows: chain *B*, light blue; chain *C*, magenta; chain *E*, pink.  (*c*, *d*) Side-by-side comparison of the Sfri0576-like family structures 2q3l (*c*) and 2ook (*d*). The equivalent nonpolar residues that are exposed on the 2q3l surface and buried in the 2ook core are shown in stick representation.

The Midwest Centre for Structural Genomics (MCSG) has determined two representative homopentameric structures of the YbjQ-like family (DUF74). In one of them (PDB entry 1y2i; J. S. Brunzelle, G. Minasov, X. Yang, L. Shuvalova, F. R. Collart & W. F. Anderson, unpublished work) the constituent subunits have identical conformations and are related by fivefold symmetry. In the other pentamer (PDB entry 1vr4; J. S. Brunzelle, L. K. McNamara, X. Yang, G. Minasov, L. Shuvalova, F. R. Collart & W. F. Anderson, unpublished work) the subunits are similarly arranged around the fivefold axis but adopt different conformations. One of the subunits has a conformation that is identical to the conformation of the 1y2i subunits, whereas a subunit on the opposite side of this pentamer has an alternative folded conformation. The central part of the latter forms a β-sheet instead of α-­helices and makes different intersubunit contacts (Figs. 7 ▶ *a* and 7 ▶ *b*). The equivalent regions in the remaining subunits of the 1vr4 pentamer are partly invisible.

Such metamorphic proteins may have evolved in other families and can account for the abrupt fold changes (Murzin, 2008 ▶; Roessler *et al.*, 2008 ▶). One recent example of this type of change was revealed by the structural comparison of two closely related proteins that are members of the Sfri0567-like family characterized by the JCSG (Kumar *et al.*, 2010 ▶). These proteins share 54% sequence identity but adopt notably different conformations referred to as ‘open’ and ‘closed’. In the ‘open’ structure (PDB entry 2q3l) two long helices define the walls of a deep groove, whereas in the ‘closed’ (PDB entry 2ook) structure these helices are refolded so that the groove is occluded (Figs. 7 ▶ *c* and 7 ▶ *d*). A substantial number of solvent-exposed nonpolar residues in the ‘open’ structure become buried in the hydrophobic core of the ‘closed’ structure. The different conform­ational states observed in 2q3l and 2ook are stabilized by the formation of different dimeric (and crystal) contacts.

## 6. Concluding remarks

One of the primary advantages of the PSI SG initiative is that it promotes discovery-driven rather than hypothesis-driven research. This approach allows the capture of many unexpected protein relationships that provide important new insights into protein-structure evolution. Structural genomics has made major contributions to the discovery of new protein topologies and architectures and thus ultimately has accelerated our understanding of protein folding.

The Protein Structure Initiative has produced a great amount of data which we are just beginning to understand and appreciate. Some of these data will also await the release of complementary information on homologous proteins and/or any experimental biological insights in order to reveal their real value. Protein structures that are single representatives of folds bear the potential for new discoveries in the near future. Their prize will be given when the structure of another representative of this fold is determined.

## References

[bb1] Andreeva, A., Howorth, D., Chandonia, J. M., Brenner, S. E., Hubbard, T. J., Chothia, C. & Murzin, A. G. (2008).* Nucleic Acids Res.* **36**, D419–D425. 10.1093/nar/gkm993 PMC2238974 18000004

[bb2] Andreeva, A. & Murzin, A. G. (2006). *Curr. Opin. Struct. Biol.* **16**, 399–408. 10.1016/j.sbi.2006.04.003 16650981

[bb3] Andreeva, A., Prlic, A., Hubbard, T. J. & Murzin, A. G. (2007). *Nucleic Acids Res.* **35**, D253-259. 10.1093/nar/gkl746 PMC1635320 17068077

[bb4] Bakolitsa, C., Bateman, A. *et al.* (2010). *Acta Cryst.* F**66**, 1198–1204.

[bb5] Bakolitsa, C., Kumar, A., Carlton, D. *et al.* (2010). *Acta Cryst.* F**66**, 1205–1210.

[bb6] Bakolitsa, C., Kumar, A., McMullan, D. *et al.* (2010). *Acta Cryst.* F**66**, 1211–1217.

[bb7] Bonneau, R., Ruczinski, I., Tsai, J. & Baker, D. (2002). *Protein Sci.* **11**, 1937–1944. 10.1110/ps.3790102 PMC2373674 12142448

[bb8] Chiu, H.-J. *et al.* (2010). *Acta Cryst.* F**66**, 1153–1159.

[bb9] Chothia, C. (1984). *Annu. Rev. Biochem.* **53**, 537–572. 10.1146/annurev.bi.53.070184.002541 6383199

[bb10] Chothia, C. & Finkelstein, A. V. (1990). *Annu. Rev. Biochem.* **59**, 1007–1039. 10.1146/annurev.bi.59.070190.005043 2197975

[bb11] Chothia, C., Hubbard, T., Brenner, S., Barns, H. & Murzin, A. (1997). *Annu. Rev. Biophys. Biomol. Struct.* **26**, 597–627. 10.1146/annurev.biophys.26.1.597 9241431

[bb12] Chothia, C., Levitt, M. & Richardson, D. (1977). *Proc. Natl Acad. Sci. USA*, **74**, 4130–4134. 10.1073/pnas.74.10.4130 PMC431889 270659

[bb13] Chothia, C. & Murzin, A. G. (1993). *Structure*, **1**, 217–222. 10.1016/0969-2126(93)90010-e 8081735

[bb14] Grishin, N. V. (2001*a*). *J. Struct. Biol.* **134**, 167–185. 10.1006/jsbi.2001.4335 11551177

[bb15] Grishin, N. V. (2001*b*). *Nucleic Acids Res.* **29**, 1703–1714. 10.1093/nar/29.8.1703 PMC31318 11292843

[bb16] Han, G. W., Bakolitsa, C. *et al.* (2010). *Acta Cryst.* F**66**, 1218–1225.

[bb17] Han, G. W., Elsliger, M.-A. *et al.* (2010). *Acta Cryst.* F**66**, 1237–1244.

[bb18] Han, G. W., Krishna, S. S. *et al.* (2010). In preparation.

[bb19] Hirano, S., Asamizu, S., Onaka, H., Shiro, Y. & Nagano, S. (2008). *J. Biol. Chem.* **283**, 6459–6466. 10.1074/jbc.M708109200 18171677

[bb20] Ho, M. C., Menetret, J. F., Tsuruta, H. & Allen, K. N. (2009). *Nature (London)*, **459**, 393–397. 10.1038/nature07938 19458715

[bb21] Kumar, A. *et al.* (2010).* Acta Cryst.* F**66**, 1245–1253.

[bb22] Levitt, M. & Chothia, C. (1976). *Nature (London)*, **261**, 552–558. 10.1038/261552a0 934293

[bb23] Lim, K., Zhang, H., Tempczyk, A., Krajewski, W., Bonander, N., Toedt, J., Howard, A., Eisenstein, E. & Herzberg, O. (2003). *Proteins*, **51**, 56–67. 10.1002/prot.10323 12596263

[bb24] Murzin, A. G. (1998). *Curr. Opin. Struct. Biol.* **8**, 380–387. 10.1016/s0959-440x(98)80073-0 9666335

[bb25] Murzin, A. G. (2008). *Science*, **320**, 1725–1726. 10.1126/science.1158868 18583598

[bb26] Murzin, A. G., Brenner, S. E., Hubbard, T. & Chothia, C. (1995). *J. Mol. Biol.* **247**, 536–540. 10.1006/jmbi.1995.0159 7723011

[bb27] Murzin, A. G., Lesk, A. M. & Chothia, C. (1994). *J. Mol. Biol.* **236**, 1382–1400. 10.1016/0022-2836(94)90065-5 8126727

[bb28] Nureki, O., Shirouzu, M., Hashimoto, K., Ishitani, R., Terada, T., Tamakoshi, M., Oshima, T., Chijimatsu, M., Takio, K., Vassylyev, D. G., Shibata, T., Inoue, Y., Kuramitsu, S. & Yokoyama, S. (2002).* Acta Cryst.* D**58**, 1129–1137. 10.1107/s0907444902006601 12077432

[bb29] Richardson, J. S. (1977). *Nature (London)*, **268**, 495–500. 10.1038/268495a0 329147

[bb30] Richardson, J. S. (1981). *Adv. Protein Chem.* **34**, 167–339. 10.1016/s0065-3233(08)60520-3 7020376

[bb31] Rife, C. *et al.* (2005). *Proteins*, **61**, 449–453. 10.1002/prot.20502 16104018

[bb32] Robinson, C. R. & Sauer, R. T. (1996). *Biochemistry*, **35**, 13878–13884. 10.1021/bi961375t 8909284

[bb33] Roessler, C. G., Hall, B. M., Anderson, W. J., Ingram, W. M., Roberts, S. A., Montfort, W. R. & Cordes, M. H. (2008). *Proc. Natl Acad. Sci. USA*, **105**, 2343–2348. 10.1073/pnas.0711589105 PMC2268138 18227506

[bb34] Sternberg, M. J. & Thornton, J. M. (1976). *J. Mol. Biol.* **105**, 367–382. 10.1016/0022-2836(76)90099-1 972389

[bb35] Sternberg, M. J. & Thornton, J. M. (1977). *J. Mol. Biol.* **110**, 269–283. 10.1016/s0022-2836(77)80072-7 845952

[bb36] Sudhamsu, J., Lee, G. I., Klessig, D. F. & Crane, B. R. (2008). *J. Biol. Chem.* **283**, 32968–32976. 10.1074/jbc.M804837200 PMC2583316 18801747

[bb37] Yooseph, S. *et al.* (2007). *PLoS Biol.* **5**, e16. 10.1371/journal.pbio.0050016 PMC1821046 17355171

